# Know your limits: RPA availability and chemoresistance in ovarian cancer

**DOI:** 10.18632/oncotarget.26607

**Published:** 2019-01-25

**Authors:** Emile Fortier, Elliot Drobetsky, Hugo Wurtele

**Affiliations:** Centre de Recherche de l'Hôpital Maisonneuve-Rosemont, Montréal, Canada; Programme de Biologie Moléculaire, Université de Montréal, Montréal, Canada; Département de Médecine, Université de Montréal, Montréal, Canada

**Keywords:** genotoxic chemotherapy, replication protein A, nucleotide excision repair, DNA replication stress, ovarian cancer

DNA replication stress (DRS), i.e. the abnormal slowing or stalling of DNA replication fork (RF) progression, is recognized as an important cause of genomic instability that in turn drives cancer development (reviewed in [1]). However, DRS is also central to cancer treatment; indeed, genotoxic anti-cancer agents including platinum compounds and nucleoside analogues selectively eliminate rapidly-dividing tumour cells by inhibiting the progression of replicative DNA polymerases. In addition, certain cancer-associated genetic aberrations, e.g, BRCA1/2 mutations in breast and ovarian tumours, have been shown to compromise the integrity of stalled RF, thereby conferring sensitivity to genotoxic chemotherapeutics [2]. The above considerations have spurred considerable research on the molecular underpinnings of DRS, with the ultimate goal of developing new biomarkers and therapeutic targets for clinical management of cancer.

Stalled RF are characterized by abnormally long stretches of single-stranded DNA (ssDNA) which become rapidly coated by heterotrimeric Replication Protein A (RPA). RPA-bound ssDNA then stages the recruitment and activation of Ataxia Telangiectasia and Rad3-related (ATR) kinase. This triggers the “intra-S phase checkpoint” phosphorylation cascade, culminating in stabilization of stalled RF and inhibition of DNA replication origin firing. Cells defective in intra-S phase checkpoint signalling, e.g. upon treatment with ATR inhibitors, fail to restrain new origin firing upon DRS, which exacerbates the consequences of RF stalling by greatly increasing the production of ssDNA. This engenders so-called “replication catastrophe” i.e., massive generation of ssDNA-RPA complexes, which exhausts the cellular pool of RPA leading to collapse of persistently-stalled RF, formation of DNA double-strand breaks (DSB), and cell death [3].

Nucleotide excision repair (NER) removes helix-destabilizing replication-blocking DNA lesions induced by a plethora of genotoxins including UV light and platinum-based chemotherapy compounds such as cisplatin (CDDP). In addition to its well-documented functions in the S phase checkpoint and DNA replication, RPA plays essential roles in lesion recognition and DNA resynthesis during NER. We originally demonstrated that abrogating the activity of either ATR or translesion DNA polymerase eta (pol eta) causes striking defects in NER of UV-induced DNA photoproducts uniquely during S phase (hereafter S phase-specific NER is referred to as SPR) [4, 5]. At the time we speculated that elevated DRS in UV-exposed ATR- or pol eta-depleted cells might lead to abnormal sequestration of RPA at stalled RF, thereby compromising the critical function of this complex in NER. In agreement, we subsequently reported that RPA overexpression rescues SPR defects in either ATR- or pol eta-deficient cells [6], and further showed using yeast that defects in multiple DRS response pathways cause both abnormal RPA accumulation on DNA during S phase and defective SPR [6]. Overall, these data indicate that several DNA damage response pathways can regulate RPA availability by influencing the frequency and/or persistence of stalled RF upon genotoxin-induced DRS.

Given the essentiality of RPA in both ATR signalling and NER, we hypothesized that modulation of its availability might influence the therapeutic efficacy of CDDP, a drug which kills cancer cells by inducing primarily DNA intrastrand crosslinks that block replication and depend on NER for their removal. CDDP is initially extremely effective for treatment of high grade serous ovarian cancer (HGSOC), but the acquisition of chemoresistance upon relapse remains a serious clinical hurdle. Very recently, our group provided novel evidence that RPA availability is a critical determinant of CDDP chemoresistance in HGSOC cells [7]. Specifically, we showed that a majority among a panel of eleven HGSOC cell lines are both deficient in SPR and profoundly sensitive to CDDP compared with SPR-proficient counterparts. Together with our previous demonstration that 11/14 model human melanoma cell lines are SPR-defective/UV hypersensitive [8], this suggested that abrogation of S phase-specific NER may represent a heretofore underappreciated feature of human cancers. We further demonstrated that overexpression of RPA restores both NER capacity and chemoresistance in SPR-defective HGSOC cells. Our data also indicated that failure to efficiently inhibit DNA replication origin firing after DNA damage contributes to RPA exhaustion in the CDDP-sensitive HGSOC cell lines that we studied.

Several recent studies have revealed important roles for the homologous recombination proteins BRCA1 and BRCA2 in forestalling nuclease-mediated degradation of nascent DNA at stalled RF, a process referred to as “RF protection” (for example see [2]). Interestingly, we found that several BRCA1/2-positive, but SPR-defective/CDDP sensitive, HGSOC cell lines among our panel present RF protection defects, indicating that reduced RPA availability might influence nascent DNA stability during DRS in a BRCA1/2-independent manner. In line with this, defective RF protection in SPR-deficient HGSOC cells could be rescued upon overexpression of RPA, suggesting that increased DSB formation during RPA exhaustion may be attributable in part to RF protection defects. Our results also suggest that reduced RPA availability during DRS may contribute to the prevalence of a “BRCAness” phenotype in HGSOC, whereby cells phenocopy BRCA1/2 deficiency even in the presence of wild-type proteins.

**Figure 1:**
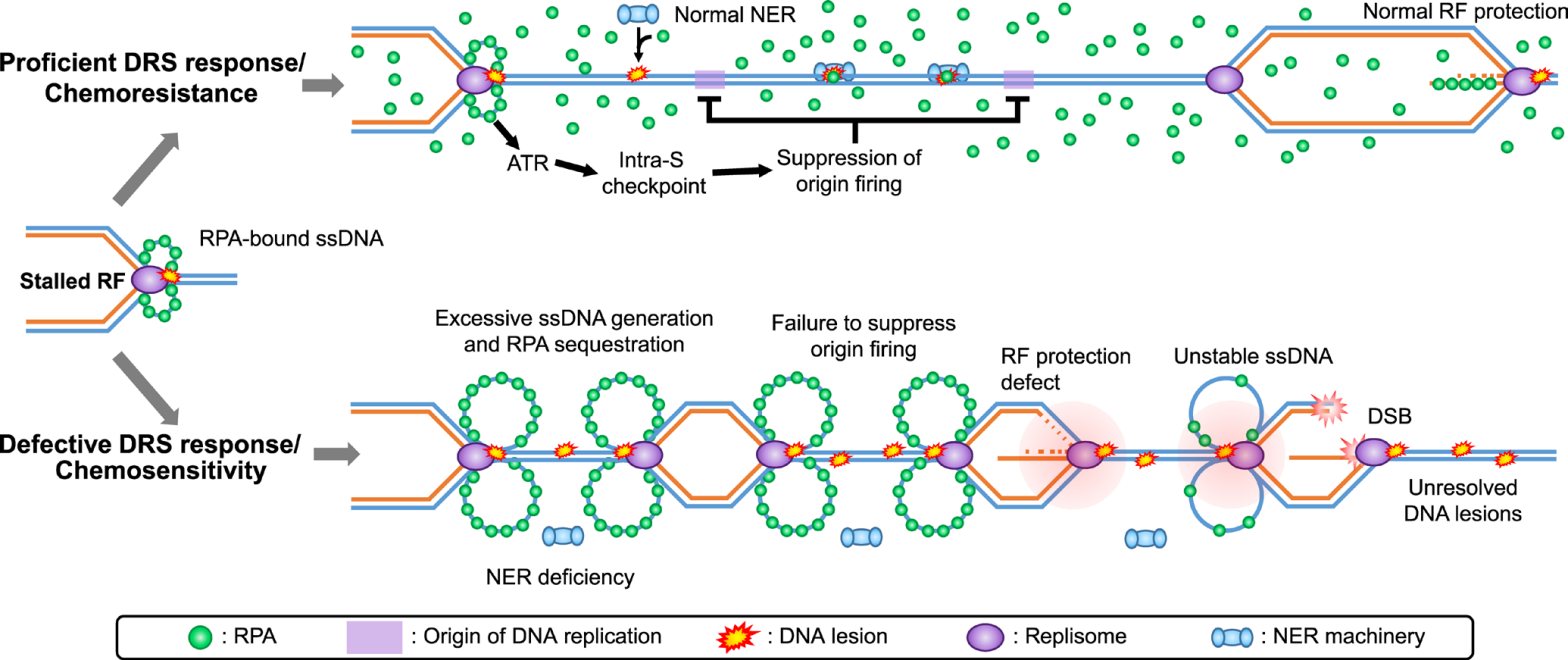
Model for DRS-mediated modulation of chemoresistance in cancer cells During genotoxic chemotherapy, a proficient DRS response (top) promotes stabilization of active RF and inhibits the firing of DNA replication origins. This in turn prevents excessive generation of ssDNA, and thus RPA sequestration, at stalled RFs. Under such conditions, chemoresistance is favoured by the availability of RPA (i) for NER which removes replication-blocking DNA lesions that relieves DRS, and (ii) for promoting adequate fork protection. In cells with a defective DRS response (bottom), failure to suppress origin firing leads to an increased number of stalled RFs and excessive formation of ssDNA-RPA complexes. RPA is therefore not readily available for either NER or fork protection, which increases DRS, replication-associated DSBs, and chemosensitivity.

Several considerations arise from our model depicting a potential role for RPA availability in chemoresistance (Figure 1). First, the emergence of RPA as a regulator of fork protection warrants further investigation. In particular RPA might influence the recruitment and/or activation of DDR factors that either protect or degrade nascent DNA at stalled RF. Second, while our results suggest that reduced RPA availability negatively impacts SPR and RF stability, the relative contributions of these latter processes to chemoresistance remains to be determined. Finally, key replication factors in addition to RPA, e.g., ones involved in replication initiation, are also present in limiting abundance in eukaryotic cells [9], and exhaustion of such factors may conceivably modulate the cellular response to DRS and thus chemoresistance. Overall, our data raise the possibility that chemoresistance in HGSOC (and other cancers) may arise at least in part via selection during genotoxic chemotherapy for variants within the tumour population with a more efficient DRS response, allowing for improved RPA availability. In addition our work lends credence to a potential therapeutic benefit for recently developed RPA inhibitors in combination with DRS-inducing drugs [10], and suggests that RPA expression levels might be predictive of chemoresistance in a variety of cancers.
